# Multiscale characterization of seawater pipe erosion of B10 copper–nickel alloy welded joints

**DOI:** 10.1038/s41598-022-06033-w

**Published:** 2022-02-09

**Authors:** Dalei Zhang, Ran Liu, Yingshuang Liu, Shaohua Xing, Liuyang Yang, Enze Wei, Xiaohui Dou

**Affiliations:** 1 School of Materials Science and Engineering, China University of Petroleum (East China), Shandong, 266580 China; 2 State Key for Marine Corrosion and Protection, Luoyang Ship Material Research Institute, Qingdao, 266237 China

**Keywords:** Fluidics, Metals and alloys

## Abstract

In seawater pipeline, the welding joint is a non-uniform structure composed of welding seam, base metal and heat affected zone. It has inhomogeneity in chemical composition, organizational structure, residual stress, etc. As local defects and high turbulence accelerate corrosion, the welding joint is often the weakest link in pipeline corrosion. Herein, the electrochemical corrosion behavior of B10 alloy welded joint in flowing seawater is studied from macroscopic and submicroscopic viewpoints using AC impedance, linear polarization, array electrode and morphological characterization. The results reveal that the corrosion rate of weld metal (WM), base metal (BM) and heat-affected zone (HAZ) decreased with the increase of time. Combined with SEM and EDS analysis, it can be seen that the increase in time led to the decomposition and accumulation of corrosion products, which gradually enhanced the corrosion resistance of welded joints. At the submicroscopic scale, WM acts as a cathode to mitigate corrosion during the later stages of high flow rate.

## Introduction

As an important component of offshore platform systems, seawater pipelines often undertake the functions of fire-fighting, cooling and heat exchange, and are used in the alternating environments of dynamic and static seawater^1–3^. The flowing seawater leads to faster diffusion of corrosive medium and accelerates the spalling of corrosion products^4–7^. Therefore, corrosion and leakage problems often occur, threatening the safety of offshore platform systems and causing serious economic and property losses^4,8–10^. In particular, the corrosion leakage of copper-nickel alloy based seawater piping system often occurs at the welded joints^11–15^. This is due to the effect of welding heat input and stress on the BM, forming a HAZ around the BM. Hence, the corrosion resistance is altered due to the differences in the composition of weld zone, base metal and heat affected zone, resulting in galvanic corrosion^16–18^. In addition, local turbulence affects the mass transfer process and alters the corrosion behavior due to weld defects and residual height^19–22^. Therefore, it is of great significance to clarify the corrosion process of Cu–Ni alloy based welded joints in pipelines from the electrochemical viewpoint^15,23–25^. Several studies have been carried out to understand the corrosion behavior of Cu/Ni alloys^26–28^. For example, Hodgkiess et al. have studied the corrosion resistance of Cu/Ni alloy in saltwater and demonstrated that the Cu/Ni alloy possesses high resistance to mechanical erosion^29^ and inferior stability towards galvanic corrosion. Yuan et al. have investigated the surface corrosion behavior of Cu/Ni alloy in simulated seawater and found that the Cu/Ni alloy in seawater forms Cu_2_O and CuO oxide film to protect the matrix and reduce the corrosion rate^30^. Ezuber et al. have studied the influence of temperature on corrosion properties of Cu/Ni alloy and found that the corrosion rate gradually increased with the increase of temperature^31^. Veena Subramanian et al. investigated the corrosion behavior of 70/30 copper-nickel alloy in potassium permanganate^32^. Cyclic polarization revealed uniform corrosion in high potassium permanganate solution. And the product film formed on the surface has a good protective effect.

In general, AC impedance spectroscopy and potentiodynamic polarization are utilized to study the corrosion behavior of different materials, including the B10 alloys. However, there is not a complete system to study the corrosion process of B10 copper and nickel alloy due to different service conditions and various influencing factors. Therefore, this paper adopts traditional electrochemical technology (EIS, LPR) and array electrode technology (WBE) are employed to investigate the electrochemical mechanism of B10 copper-nickel alloy welded joint under flowing seawater^33–37^, and combined with surface characterization (SEM and EDS) techniques to understand the corrosion behavior at macro and sub-micron scales. In general, AC impedance spectroscopy and potentiodynamic polarization are utilized to study the corrosion behavior of different materials, including the B10 alloys. However, there is not a complete system to study the corrosion process of B10 copper and nickel alloy due to different service conditions and various influencing factors. Therefore, this paper adopts traditional electrochemical technology (EIS, LPR) and array electrode technology (WBE) are employed to investigate the electrochemical mechanism of B10 copper-nickel alloy welded joint under flowing seawater^33–37^, and combined with surface characterization (SEM and EDS) techniques to understand the corrosion behavior at macro and sub-micron scales. In this paper, the corrosion behavior of three zones of B10 alloy welded joint under different flow rates is analyzed from different scales, and the galvanic corrosion resistance of the whole welded joint under different flow rates is compared. The corrosion behavior of B10 alloy welded joint is described in detail. It provides reliable reference for corrosion failure behavior of copper-nickel alloy seawater pipe.

## Experimental section

### Macro-electrode material

**Figure 1:**
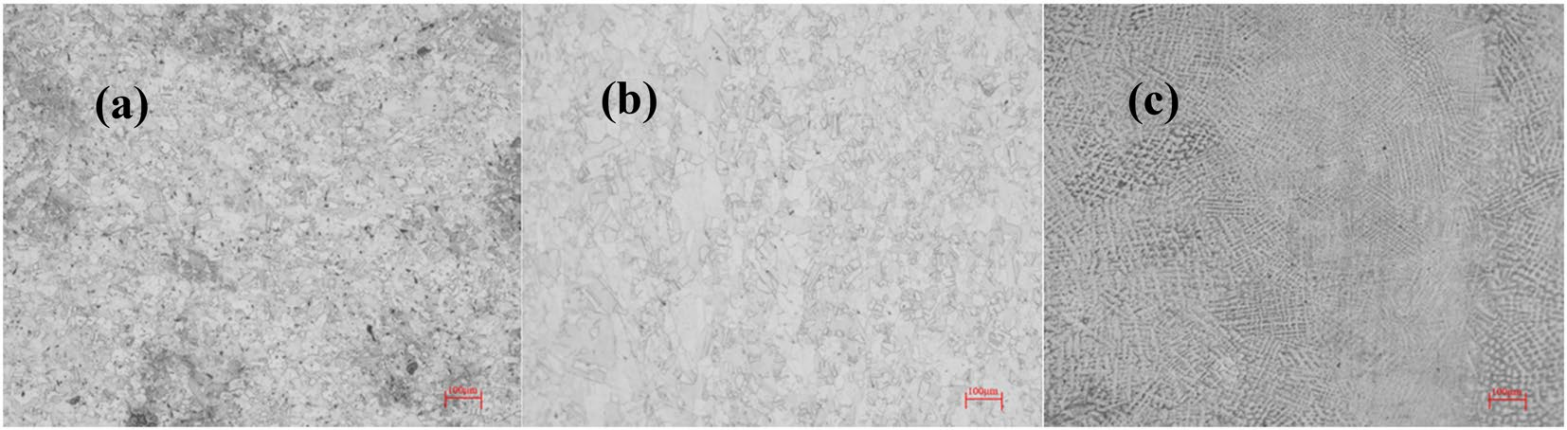
Microstructure of B10 Cu–Ni alloy: (**a**) BM, (**b**) HAZ, (**c**) WM.

Domestic B10 pipe material (BM) and TIG welding seam (WM, wire grade HSCuNi) and heat-affected zone (HAZ) material were analyzed. Figure 1 shows the metallographic photos of three zones of B10 copper nickel alloy welded joint taken by ZEISS microscope. It can be seen that the microstructure of the WM is uniform and dense without defects. There are many austenite phases in the BM, and the microstructure is uniform and equiaxed. However, compared with the BM, the HAZ is a coarse austenite phase. This is related to the influence of heat source input on welding. The corresponding chemical composition is shown in Table 1. It adopts direct reading spectrometer, model Q4 Tasman 130. The three sections of the welded joint were machined into square samples of 10 mm × 10 mm × 5 mm by mechanical processing. The backside was connected with copper wire and, then, the samples were placed in a mold for encapsulation with epoxy resin. Prior to the experiment, 240–1200 molybdenum water sandpaper was used for polishing, whereas pure water and anhydrous ethanol were used for cleaning and blow-drying.

**Table 1:** The composition of B10 copper-nickel alloy welding joint.

Material	Main ingredients				Impurities (≤)					
	Ni	Fe	Mn	Cu	Si	S	C	Zn	P	Other impurities
Parent metal	10.28	1.59	0.86	87.1	0.038	0.004	0.024	0.003	0.006	0.095
Welding materials	24.29	0.79	0.82	73.6	0.016	0.005	0.012	0	0.007	0.46

### Submicroscopic electrode materials

**Figure 2:**
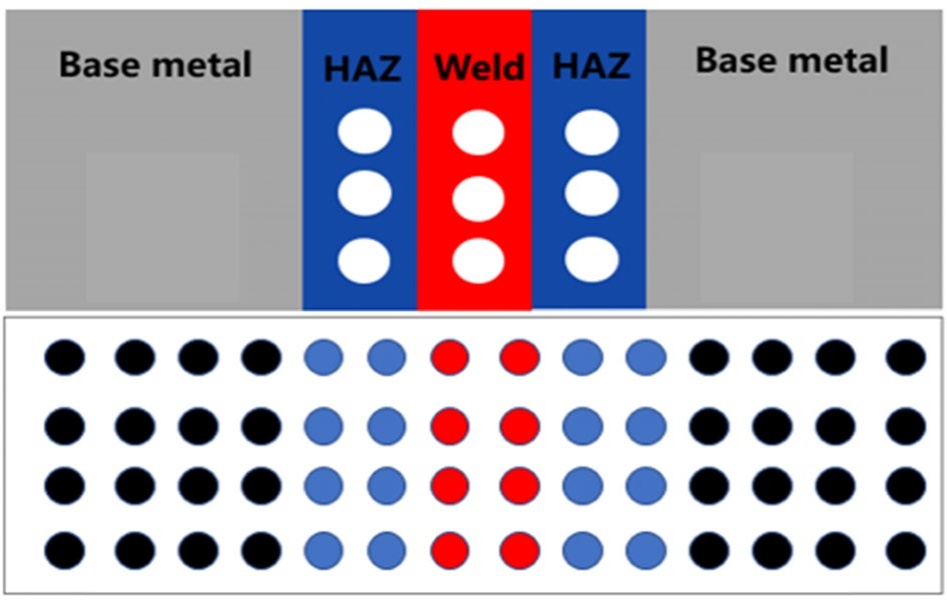
Arrangement of array electrode wires.

First, the B10 welded joint was processed into electrode wires with a diameter of 1 mm and a length of 50 mm. The electrodes were installed into the designed slots according to the area ratio of three sections and the spacing of electrode wires was 0.7 mm, as shown in Fig. 2. Black represents BM, blue represents HAZ, and red represents WM. The machined electrode wire was ultrasonically cleaned with anhydrous ethanol to remove the oil, polished with sandpaper and, then, put into the designated slot. In order to make the electrode insulated, the surface was evenly sprayed with three-proof paint and, then, the coating was evenly placed into the mesh and encapsulated with epoxy resin. After the epoxy resin was solidified, the surface was polished with water and sandpaper. After cleaning with pure water, a multi-meter was used to measure the conductivity between electrode and row wires.

### Experimental equipment

The artificial seawater was used as the experimental medium, as detailed in Table 2.

**Table 2:** Concentration table of artificial seawater preparation.

Composition	NaCl	MgCl_2_·6H_2_O	MgSO_4_·7H_2_O	CaCl_2_	KCl	NaHCO_3_	NaBr	Cl^−^ wt.%
Concentration (%)	5.34	0.52	0.67	0.11	0.072	0.02	0.008	3.5

**Figure 3:**
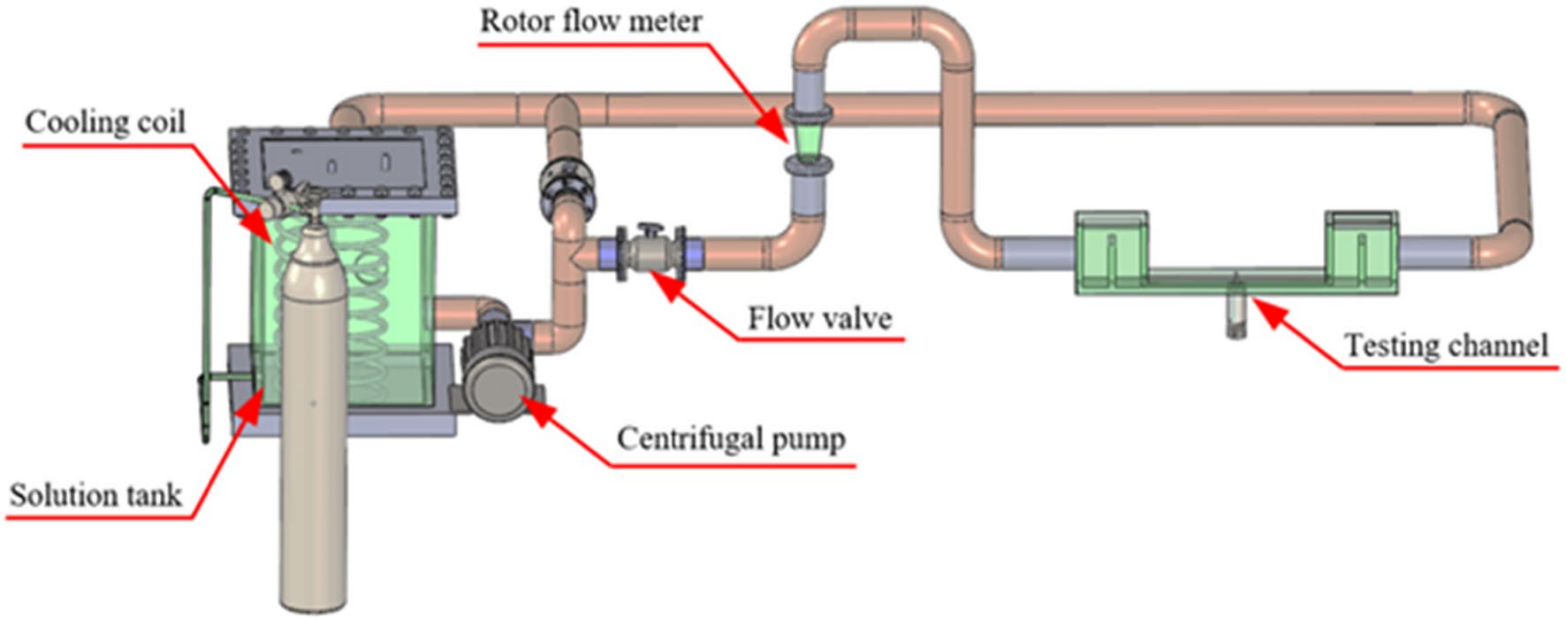
The unidirectional flow accelerated corrosion test circuit.

**Figure 4:**
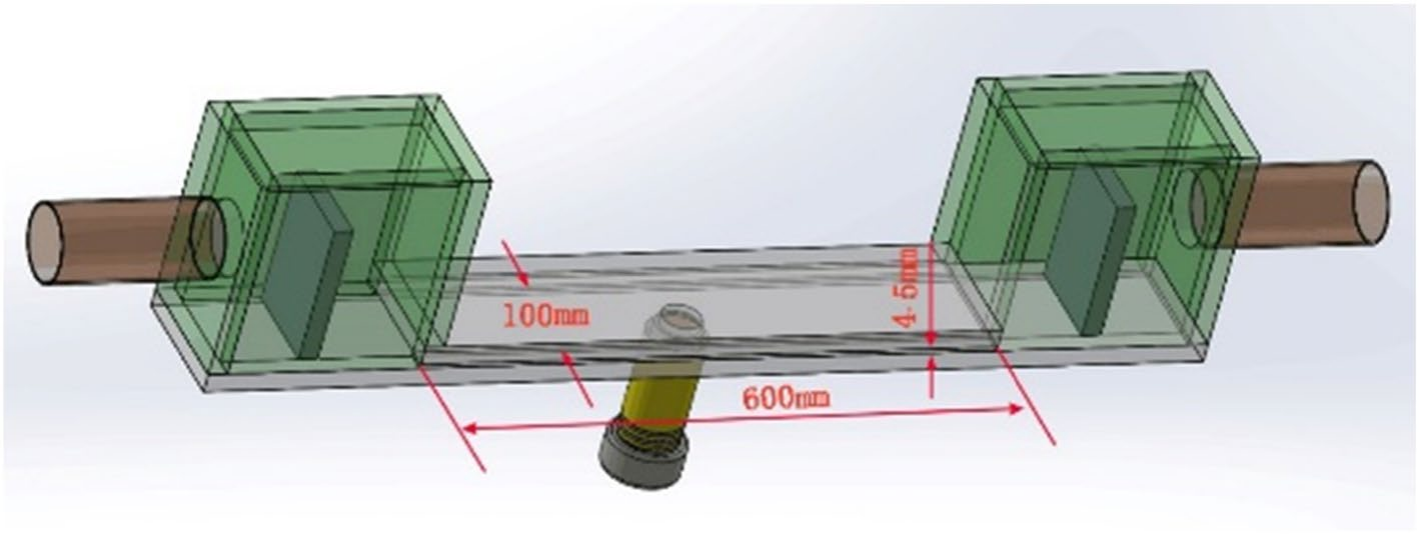
The testing channel of the flow-accelerated corrosion loop system.

The experimental equipment adopted the one-way flow accelerated corrosion test circuit, which was designed by our team. The flow corrosion test circuit consisted of a solution box, a centrifugal pump, a control valve, a flow channel and a flow meter. The device can simulate the erosion conditions of seawater pipelines, whereas the flow channel can calculate the cross-sectional area and flow per unit time to estimate the actual flow rate, which can be controlled by the control valve (0–6 m/s). The artificial seawater was stored in a solution box and pumped into the pipeline through a centrifugal pump. As the temperature increases due to the solution flow, a cooling coil was placed in the solution box and cold water was injected during the experiment to control the temperature. The schematic illustration of the device is shown in Fig. 3 and the test area is shown in Fig. 4.

### Experimental solution

A three-electrode system was used to measure the electrochemical corrosion behavior during the macroscopic experiment. Three sections of the B10 copper–nickel alloy welded joint were used as the working electrode (WE), platinum wire as the counter electrode (CE), and high-purity zinc as the reference electrode (RE). The relative standard hydrogen potential was − 0.762 V (vs. ocp). The model of electrochemical workstation is Solartron1287 + 1255B corrosion electrochemical test system.

**Figure 5:**
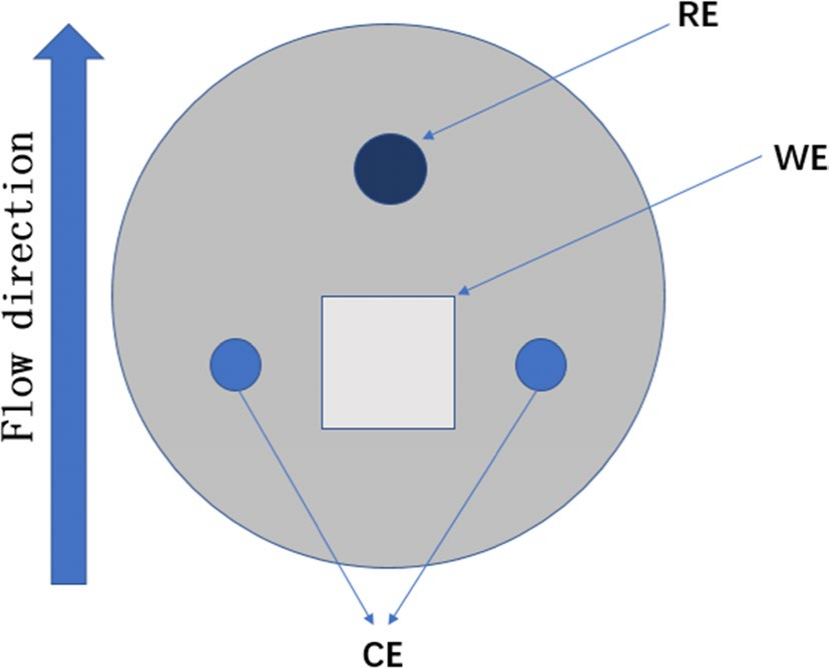
The macroscopic arrangement of the three-electrode setup.

The specific arrangement of three electrode system is shown in Fig. 5. Before the experiment begins, the three-electrode systemwas encapsulated in epoxy resin and loaded into the experimental channel. The open-circuit potential, electrical impedance and linear polarization curves at different times were measured every 1 h at different flow rates. The frequency range of AC impedance spectroscopy was 100 kHz to 0.01 Hz, and the amplitude of AC excitation signal was 5 mV. The linear polarization scanning rate was 10 mV/min and the scanning range was ± 15 mV (vs. OCP). Three groups of repeated experiments were carried out for all corrosion experiments, and the results were obtained.

**Figure 6:**
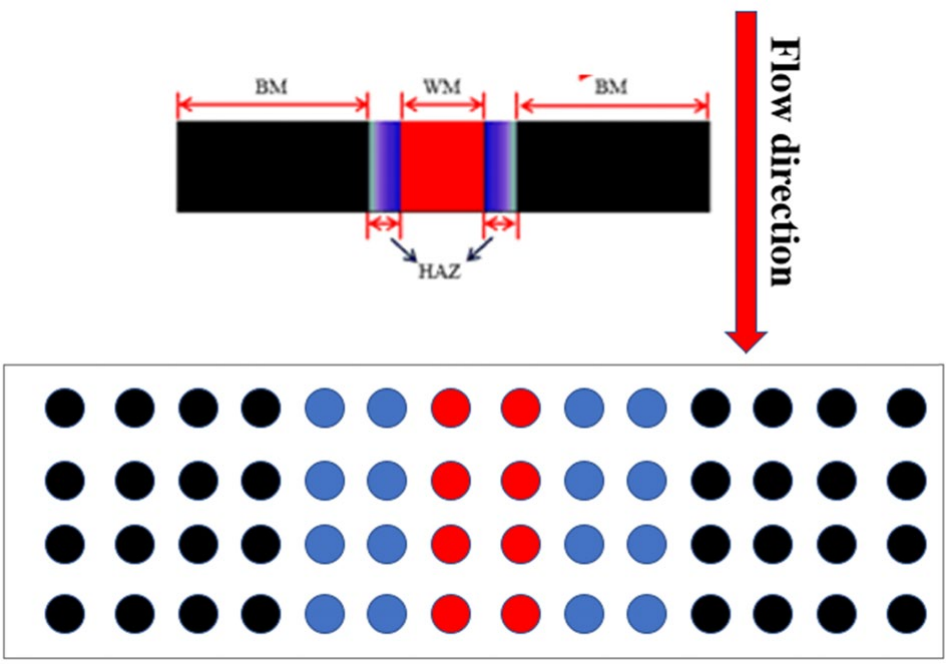
Flow test arrangement of the array electrode.

Furthermore, the array electrode technology was used during the submicroscopic experiments, as shown in Fig. 6. The model of array electrode test equipment is NI PXI microelectrode array automatic detection system. When testing the galvanic current, the galvanic pair must be in the short-circuit state, which could be realized by connecting the electrode wires to the pXI-4071 and PXI-4022 measuring plate in the established order and separating the electrode wires from the electrode array. Hence, a galvanic couple is formed between the electrode wire and electrode array. Furthermore, all the remaining electrode wire ports were sequentially connected to measure the galvanic current. To measure the potential, the electrodes were disconnected and the electrode potential was measured against the reference electrode.

### Surface topography

**Figure 7:**
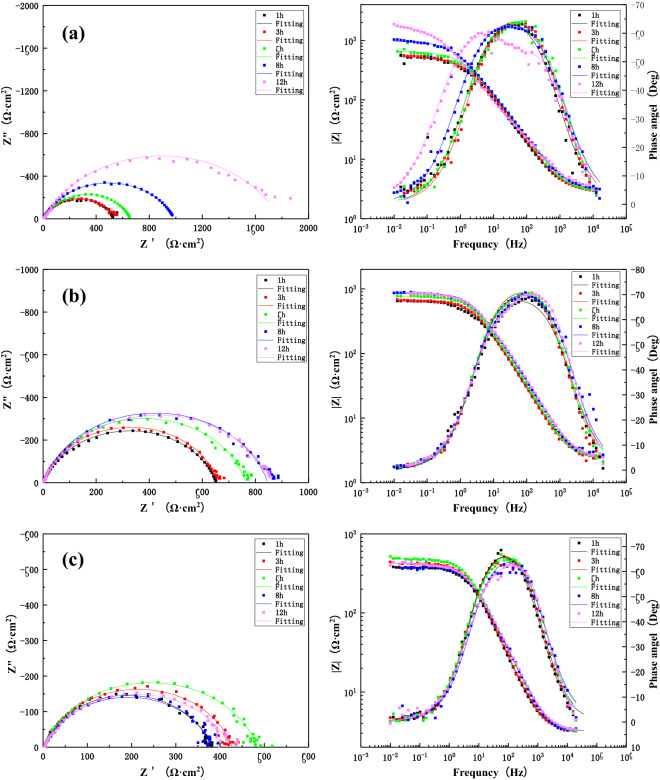
The change in impedance of the BM after 12 h scouring in seawater with three different flow rates: (**a**) 1.85 m/s, (**b**) 3 m/s, (**c**) 6 m/s.

After the corrosion experiment, the sample surface was washed with pure deionized water. After drying, scanning electron microscopy was used to observe the surface morphology of the three zones at different flow rates for 12 h after scouring. SEM equipment model (FEI QUANTA FEG2500). Moreover, EDS (Oxford Inca Energy X-Max-50) was used to analyze the composition of surface products, explaining the corrosion process and mechanism by identifying the corrosion products (Fig. 7).

## Results and discussion

### EIS analysis

**Figure 8:**
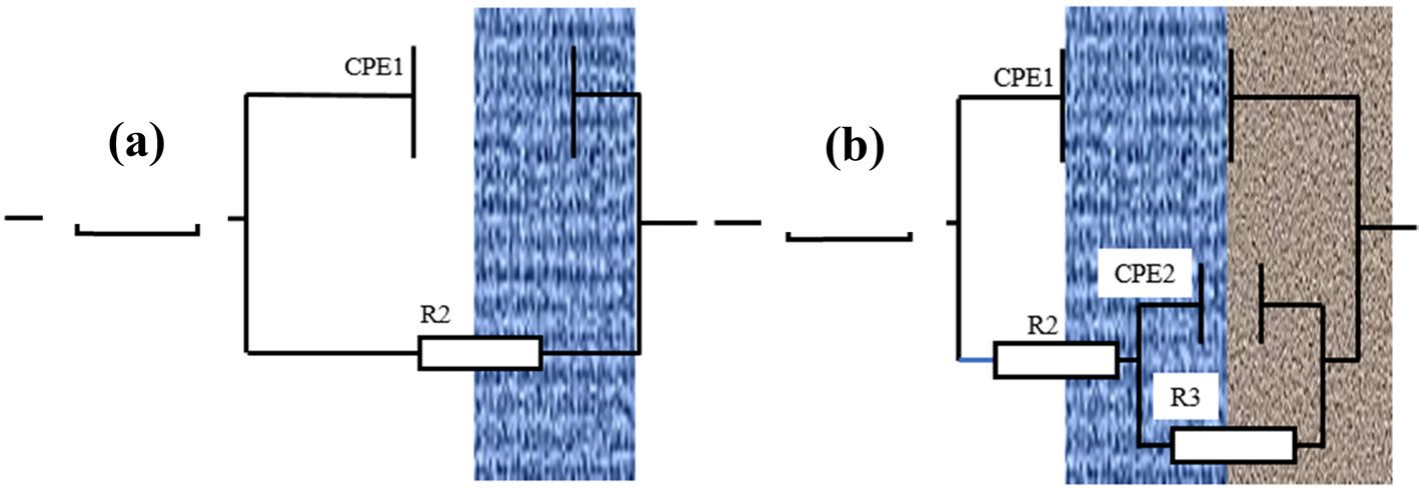
The equivalent circuit model used to fit the impedance data.

The equivalent circuit was used for fitting the impedance spectra (Fig. 8). In (a), R1 represents the solution resistance, CPE1 represents the electric double-layer capacitance of the interface between B10 alloy sample and seawater, and R2 represents the total resistance of the electrode interface. In (b), R1 represents the solution resistance, CPE1 represents the capacitance of surface film, R2 represents the resistance of surface film, CPE2 refers to the electrical double layer capacitance of the electrode/solution interface, and R3 denotes the charge transfer resistance of the sample. As the actual test conditions are different from the idealized model, the constant phase element (CPE) was used to replace the non-idealized capacitance element. When B10 alloy was corroded under flowing seawater, Cu_2_O corrosion product film was formed to protect the substrate^38,39^. The following reactions often occur:

1 $${\text{Anodic}}\,{\text{reaction}}:{\text{Cu}} + {\text{2Cl}}^{ - } \to {\text{CuCl}}_{{2}}^{ - } {\text{ + e,}}$$

2 $${\text{2CuCl}}_{{2}}^{ - } {\text{ + H}}_{{2}} {\text{O}} \to {\text{Cu}}_{{2}} {\text{O + 2H}}^{ + } {\text{ + 4Cl}}^{ - } ,$$

3 $${\text{Cathode}}\,{\text{reaction}}:{\text{O}}_{{2}} + {\text{2H}}_{{2}} {\text{O}} + {\text{4e}} \to {\text{4OH}}^{ - } .$$

Therefore, R is taken as the total resistance, where R = R2 + R3 represents the sum of product film resistance and charge transfer resistance.

Figure 7 shows the Nyquist and Bode plots of the BM area at the welding joint of B10 alloy under flowing seawater at the speed of 1.85 m/s, 3 m/s and 6 m/s for 12 h. Through Fig. 7 equivalent circuit fitting the coma now under three kinds of velocity of flow corrosion of parent metal area in low-frequency area are characterized by large capacitive reactance arc on behalf of the forming process of product. However, the seawater flow leads to the oxidation of the outer loose product off so only reaction in a single layer information, in the late stage of corrosion will appear in high-frequency area small capacitive reactance arc on behalf of the charge transfer process. Overall, the arc resistance of the BM area increased at three flow rates of 1.85 m/s, 3 m/s and 6 m/s, and the impedance reached the maximum at 1.85 m/s after 12 h. This is due to the improvement in O_2_ and other reducing agents, which facilitated the anodic reactions and charge transfer and mass transfer processes, increasing the corrosion rate and promoting the rapid formation of the surface film. The gradual accumulation of the surface film can better protect the matrix. Hence, the impedance value is gradually increased and the corrosion rate is reduced. However, at 6 m/s, the high flow velocity of shear force due to the increase in water flow velocity destroyed the surface film, compromising the integrity of protective layer. The stripping of the product film increases the corrosion rate. One should note that the higher mass transfer rate and rapid corrosion are expected to generate a new surface film to protect the matrix.

**Figure 9:**
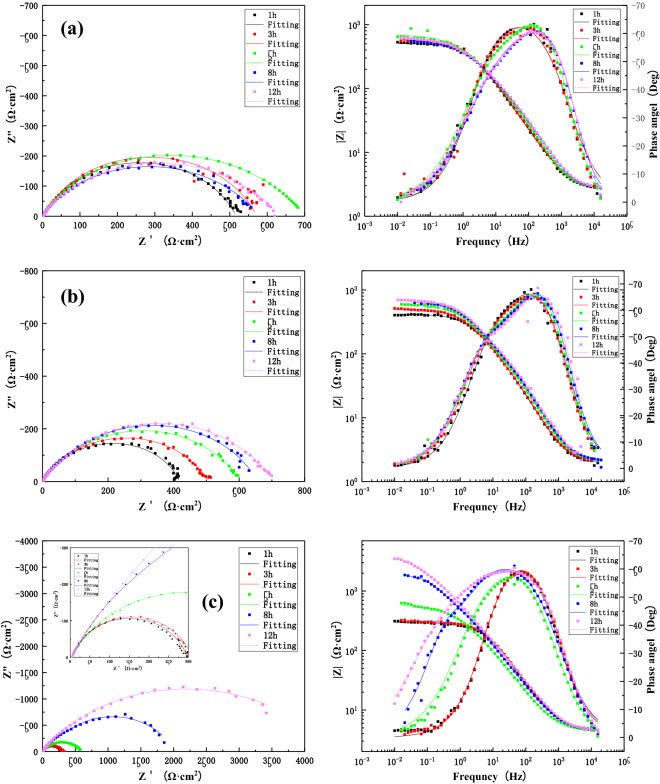
The impedance changes of the HAZ during 12 h scouring in seawater with different flow rates: (**a**) 1.85 m/s, (**b**) 3 m/s, (**c**) 6 m/s.

Figure 9 presents the Nyquist and Bode diagrams during the process of 12 h scouring of the HAZ at 1.85 m/s, 3 m/s and 6 m/s under the condition of seawater corrosion. It can be seen that the Nyquist plots consist of a single capacitance-reactance arc. After equivalent circuit fitting (Fig. 7), it can be concluded that the capacitance–reactance arcs exhibit an increase under 3 m/s and 6 m/s. Also, a significant increase of capacitance-reactance arc can be observed at later stages of 6 m/s. However, at 1.85 m/s, there was an obvious decline till 8 h, followed by an upward trend. The reason is that the corrosion products are accumulated on the surface under flowing seawater, rendering a protective effect. One should note that the HAZ is formed during the welding process due to heat input and other effects on the BM structure. However, the structure is not dense enough and exhibits significant deviations in the proportion of different components. Therefore, it is easier to produce the shredding of components and stripping of the product film during the scouring process. However, the relatively denser product film is retained within the matrix and the repeated formation of product film exhibits a strong binding force, protects the matrix, and improves the corrosion resistance^40,41^. However, the cost of this phenomenon is additional component dissolution and product spalling, which is observed under the corrosion condition of 6 m/s. At 1.85 m/s, the stripping and re-formation of the product film at a low flow rate are much slower than the high flow rate. Hence, the impedance values initially decrease with the stripping of the product film, followed by a gradual increase with the formation of the new product film. This process is similar to the formation of a denser product film due to repeated cycling.

**Figure 10:**
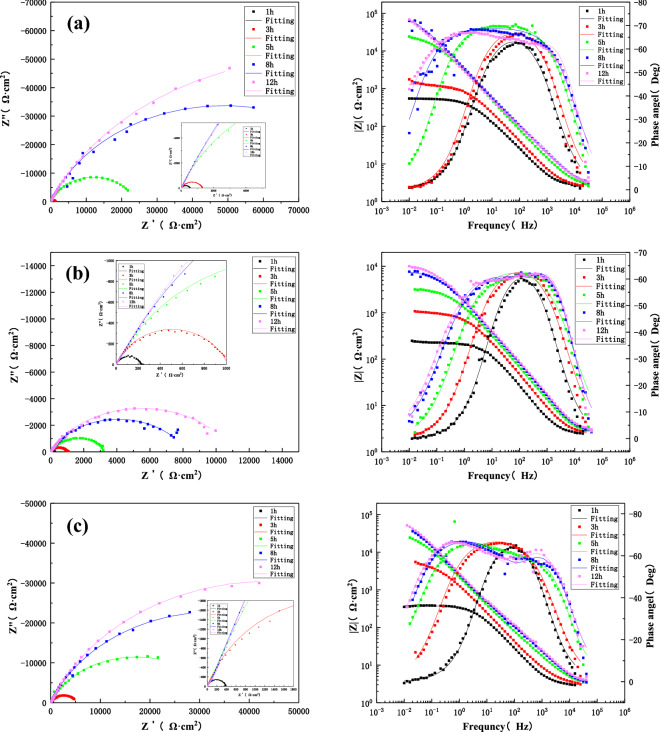
The impedance changes in the WM during 12 h scouring in seawater with three different flow rates: (**a**) 1.85 m/s, (**b**) 3 m/s, (**c**) 6 m/s.

Figure 10 presents the Nyquist and Bode diagrams during 12 h of the corrosion process of the WM of the B10 Cu/Ni alloy welded joint under three different flow rates (1.85 m/s, 3 m/s and 6 m/s). It can be seen that the WM is composed of a high-frequency capacitive arc and middle- and low-frequency capacitive arcs, where the high-frequency capacitive arc represents the charge transfer process and the middle- and low-frequency capacitive arcs represent the formation of surface film. According to the fitting results of equivalent circuit model in Fig. 7, it is found that the arc reactance of WM increases with the increase of flow scouring time at three flow rates. The reason is that the water flow during the scouring process leads to a higher rate of mass transfer, accelerating the diffusion and transmission of oxygen and corrosive medium, increasing the corrosion rate and forming the corrosion product film. In addition, the microstructure of the weld material is found to be more dense than the BM and HAZ due to the high Ni content and a near-perfect Cu–Ni ratio^41–43^. Therefore, the resulting corrosion product film is more dense, which renders better protection and improves corrosion resistance. It is noteworthy that the maximum impedance value reached 10^5^ orders of magnitudes of what after 12 h of flowing seawater under 1.85 m/s, whereas the corresponding value under 3 m/s was found to be minimum. Hence, the film forming effect of the WM is more obvious at 1.85 m/s.

**Figure 11:**
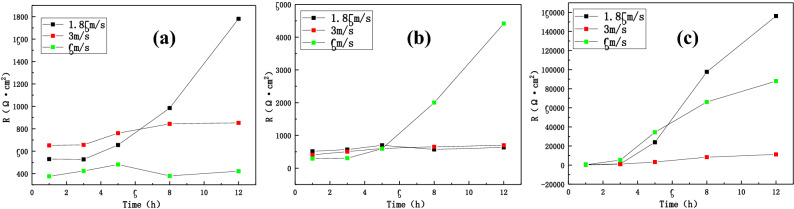
The comparison of total impedance values of three zones of welded joints under different flow rates: (**a**) BM, (**b**) HAZ and (**c**) WM.

Figure 11 shows the comparison of the fitted total impedance values (R) of three zones of the B10 alloy welded joint under different flow rates. It can be seen that the impedance value of the BM area decreased with the increase of flow rate after 12 h of corrosion. Hence, the total impedance followed the given trend: 1.85 m/s > 3 m/s > 6 m/s. In the HAZ, the impedance value increased with the increase of flow rate and the total impedance value followed the given trend: 6 m/s > 3 m/s > 1.85 m/s. One should note that the weld is more sensitive to the change in flow rate, reaching an impedance of 10^5^ orders of magnitude higher at 1.85 m/s than WM and HAZ, which is inseparable from material composition and density of tissue. Overall, at the same flow rate, the total impedance value of the three zones of welded joint followed the given trend: WM > BM > HAZ. Hence, the weld exhibits good corrosion resistance and the maximum corrosion rate of HAZ becomes the weakest point of the welded joint.

### LPR measurements and analysis

**Figure 12:**
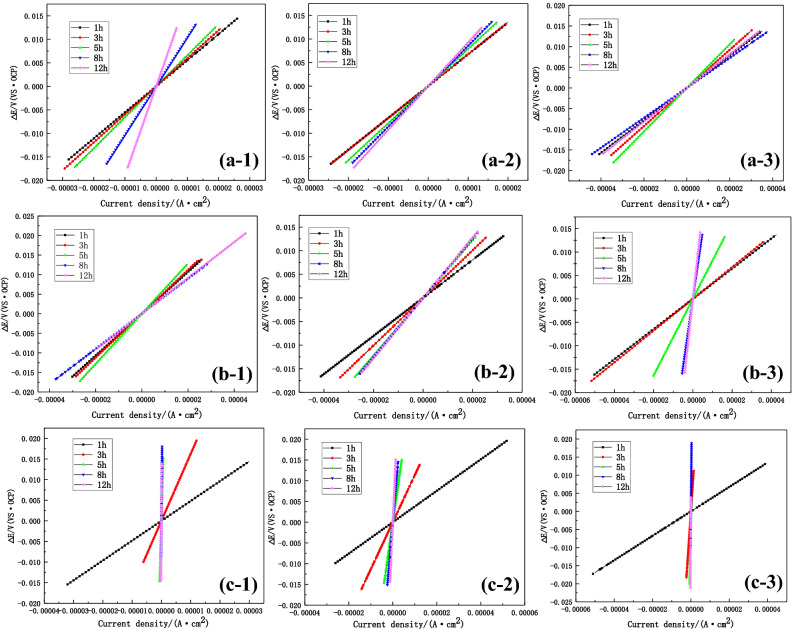
LPR curves of three zones of B10 alloy welded joint at 1.85 m/s, 3 m/s and 6 m/s after 12 h of flowing seawater corrosion: (**a**) BM, (**b**) HAZ, (**c**) WM, (1) 1.85 m/s, (2) 3 m/s, and (3) 6 m/s.

Figure 12 shows the linear polarization curves of the BM, HAZ and weld material during 12 h of flowing seawater at different flow rates of 1.85 m/s, 3 m/s and 6 m/s. The corrosion rate can be calculated by the Stern–Geary and Faraday's laws^44^:

4 $$i_{coor} = \frac{1}{{R{\text{p}}}} \cdot \frac{{{\text{b}}_{{\text{a}}} {\text{b}}_{{\text{c}}} }}{{2.303({\text{b}}_{{\text{a}}} + {\text{b}}_{{\text{c}}} )}},$$

5 $$V_{corrosionrate} = \frac{icoor \cdot A}{{{\text{n}} \cdot F \cdot \rho }},$$

where b_a_ and b_c_ denote the constant involved in the corrosion process, which are determined by the anodic and cathodic slopes of the Tafel curves, *i*_coor_ refers to the corrosion current density, *F* represents the Faraday constant (96,500 C/mol), *A* refers to the atomic mass of Fe (56), *n* represents the number of transferred electrons, and *ρ* corresponds to the density of iron.

**Figure 13:**
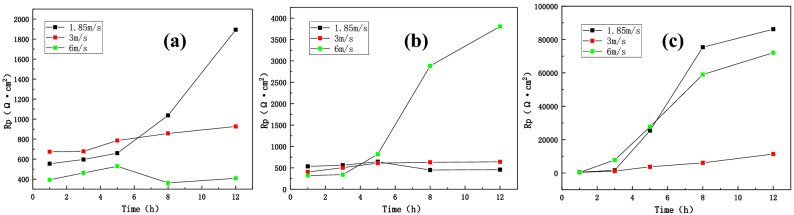
LPR comparison of three sections of B10 alloy welded joint at different flow rates: (**a**) BM, (**b**) HAZ, and (**c**) WM.

The fitted polarization resistance data are shown in Tables 3, 4 and 5. At 1.85 m/s and 3 m/s, the polarization resistance of the BM increased and the corrosion rate decreased with the extension of time. At 6 m/s, what decreased during 8 h, followed by an increase. In the case of HAZ, at 1.85 m/s, the polarization resistance decreased during 8 h, whereas the polarization resistance significantly increased at 3 m/s and 6 m/s, indicating a lower corrosion rate. However, the weld area only exhibited the increasing trend under three flow rates and reached an extremely high resistance value at 1.85 m/s. Figure 13 compares the results of three zones of B10 welded joint under different flow rates, showing excellent consistency between LPR and impedance analyses.

**Table 3:** The fitting data of polarization resistance in the BM region.

Velocity (m/s)	1 h	3 h	5 h	8 h	12 h
1.85	554.26	596.05	659.7	1037	1893.2
3	674.1	677.43	785.85	856.29	926.25
6	393.08	461.83	528.08	362.21	407

**Table 4:** The fitting data of polarization resistance in the HAZ.

Velocity (m/s)	1 h	3 h	5 h	8 h	12 h
1.85	534.45	560.56	641.92	448.89	458.04
3	403.1	503.93	609.49	629.39	639.28
6	322.29	338.8	817.26	2883.9	3804

**Table 5:** The fitting data of polarization resistance in the WM.

Velocity (m/s)	1 h	3 h	5 h	8 h	12 h
1.85	481.23	1624.5	25,387	75,387	86,180
3	378.27	1133.5	3693.4	6089.2	11,350
6	337.62	7760.8	27,649	59,003	72,095

### SEM characterization

**Figure 14:**
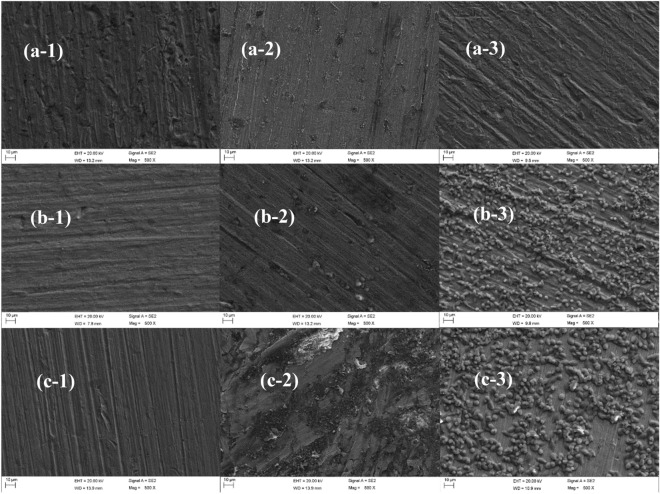
SEM images of three zones under flowing seawater for 12 h: (**a**) BM, (**b**) HAZ, (**c**) WM, (1) 1.85 m/s, (2) 3 m/s, and (3) 6 m/s.

Figure 14 shows the surface topography of the three zones of B10 alloy welded joint scoured and corroded by flowing seawater for 12 h under the flow rate of 1.85 m/s, 3 m/s and 6 m/s. It can be seen that the surface of BM area is relatively smooth at 3 m/s, and there are corrosion pits at 1.85 m/s and 6 m/s. According to EDS analysis in Table 6, the Cu–Ni ratio of normal B10 alloy is 9:1^41^, but the Cu–Ni ratio is significantly lower than 9:1 in the three flow states, indicating that the consumption of Ni element is high when the product film is formed in the flow corrosion state. The surface of the HAZ is smooth at 1.85 m/s and some granular products are seen at 3 m/s. Based on the density of granular products under the condition of 6 m/s and EDS analysis (Table 7), the content of Ni element in the membrane is far above the normal Ni content in matrix and shows that under the condition of 6 m/s corrosion Ni element from the larger, and impedance analysis are consistent, so that under 6 m/s because Ni elements from more, The product film stripping is more intense, so it has better corrosion resistance. The surface of the WM is relatively smooth at 1.85 m/s, indicating that the corrosion product film is easily formed and the impedance value is around 10^5^ orders of magnitude higher than BM and HAZ. At 3 m/s, the obvious peeling of corrosion product film can be seen, which is consistent with the lowest impedance of the WM at 3 m/s. At 6 m/s, there is a layer of corrosion products uniformly covering the matrix. The Cu–Ni ratio in the WM is normally 7:3. According to the EDS analysis (Table 8), the Cu–Ni ratio is about 2:1 after 12 h scouring, which is the same as complete substrate, indicating that the formation of the product film is due to uniform dissolution of Cu/Ni alloy during the corrosion process. One should note that this is different from the BM and HAZ.

**Table 6:** The elemental composition of BM of B10 alloy under different seawater flow rates.

Velocity (m/s)	C	O	Cl	Mg	Mn	Fe	Ni	Cu
1.85	4.37	53.90	0.76	6.39	0.35	1.70	5.57	26.96
3	4.14	30.19	0.54	3.06	0.67	2.21	8.48	53.78
6	4.61	38.24	0.62	0.90	0.55	2.92	8.52	43.63

**Table 7:** The elemental composition of the HAZ of B10 alloy under different seawater flow rates.

Velocity (m/s)	C	O	Cl	Mg	Mn	Fe	Ni	Cu
1.85	3.72	57.11	0.57	1.42	0.25	2.74	7.59	26.6
3	7.08	35.23	–	1.67	0.71	2.46	9.63	43.48
6	5.24	57.47	4.42	3.03	2.65	3.05	7.38	15.74

**Table 8:** The elemental composition of B10 alloy WM under different seawater flow rates.

Velocity (m/s)	C	O	Cl	Mg	Mn	Fe	Ni	Cu
1.85	5.27	33.22	0.68	2.75	0.58	0.91	16.61	39.98
3	5.84	34.32	0.69	3.06	0.59	1.18	17.08	37.25
6	3.33	32.30	–	3.17	0.71	0.63	17.82	42.40

### WBE results

**Figure 15:**
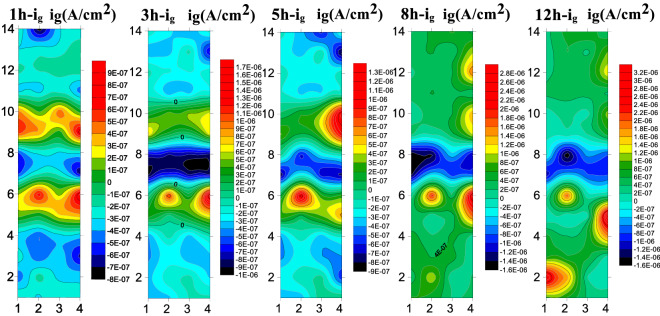
The array electrode results of B10 alloy welded joint area at 1.85 m/s.

Figure 15 presents the test results of B10 alloy welded microarray electrode at 1.85 m/s. The cathodic current density of the BM is always the cathode current during 1–5 h, and gradually decreases with the prolongation of corrosion time and becomes anodic current after 5 h. Also, the anodic current density gradually increases with the prolongation of scouring time. The anodic current is always present in the HAZ and the anodic current density gradually increases with the increase of scouring time, showing a more obvious corrosion tendency. During whole 12 h of scouring, the WM exhibited cathodic current and always acted as cathode. By comparing the corrosion current density of the three zones, it could be shown that the WM exhibited the slowest corrosion process.

**Figure 16:**
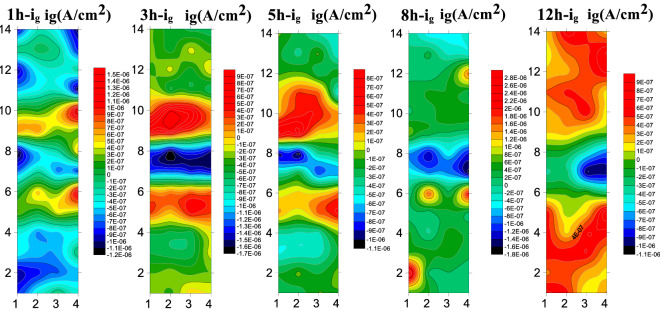
Array electrode test results of B10 alloy welded joint area at 3 m/s.

Figure 16 presents the results of submicroscopic array electrodes measured for the B10 alloy welded joint during 12 h scouring under the flow rate of 3 m/s. At 3 m/s, the weld still acts as a cathode during the whole scouring process, which is manifested as the cathodic current density. Also, the cathodic current density gradually decreased with the increase of scouring corrosion time, showing better corrosion resistance. The anodic current density was always observed in the HAZ and the anodic current density firstly increased with the increase of scouring process, followed by a gradual decrease indicating that the formation of product film renders a certain influence on the HAZ. During the whole flow corrosion process, the BM area still acts as a cathode to slow down the corrosion during 1–5 h, but the polarity starts to shift from cathode to anode to accelerate the corrosion after 8th h.

**Figure 17:**
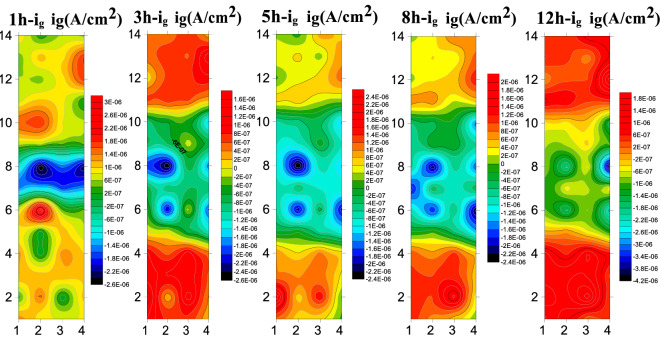
The array electrode test results of B10 alloy welded joint area at 6 m/s.

Figure 17 presents the measured submicroscopic array electrode of the B10 alloy welded joint during 12 h of scouring at 6 m/s. It can be seen that there are obvious corrosion differences between different zones of welding joints during the early stage of flow corrosion. The WM still presents cathodic current at a high flow rate of 6 m/s, maintaining excellent corrosion resistance. During the initial stage of flow corrosion, the HAZ exhibits anodic current, but it changes into the cathodic current with the progress of flow corrosion. Under the action of high flow velocity, the structure of HAZ is not dense enough, and the shredding of components and products is more than enough to demonstrate corrosion resistance. At 6 m/s, the base material area is more uniform in composition and denser in structure, resulting in slower decomposition corrosion, insufficient product film binding force and relatively poor corrosion resistance. Therefore, it manifests anodic current density and accelerates the corrosion process as an anode.

**Figure 18:**
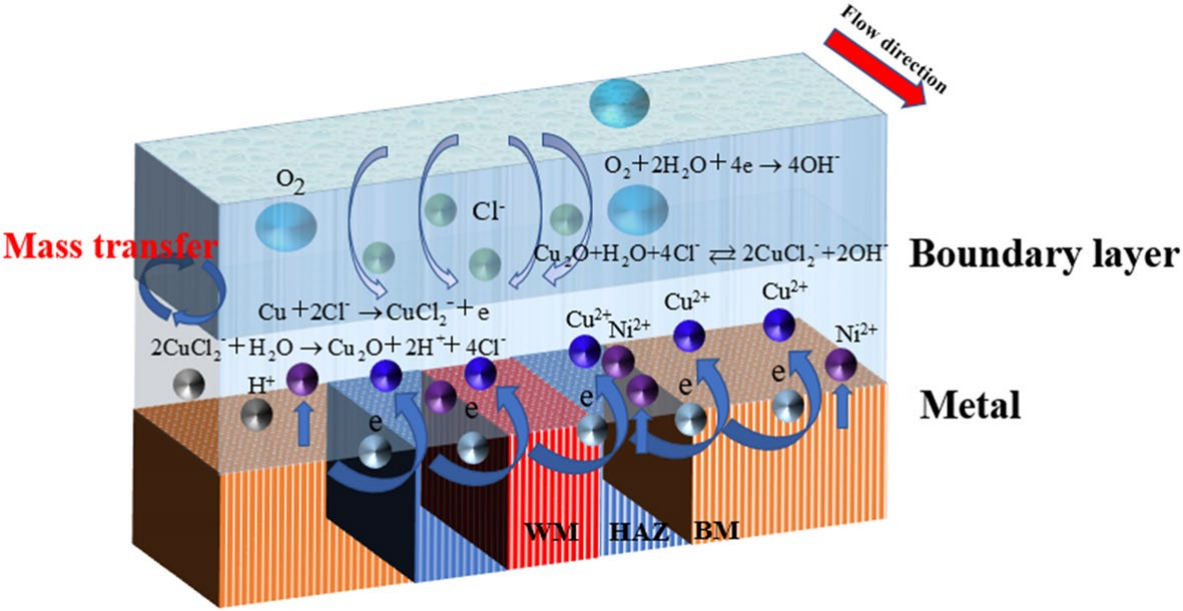
Corrosion reaction process of B10 alloy welded joint in flowing seawater.

Figure 18 shows the schematic diagram of the corrosion process of B10 copper-nickel alloy welding joint area in flowing seawater. The main reactions in this process are as follows:

6 $${\text{O}}_{{2}} + {\text{2H}}_{{2}} {\text{O}} + {\text{4e}} \to {\text{4OH}}^{ - } ,$$

7 $${\text{Cu}} + {\text{2Cl}}^{ - } \to {\text{CuCl}}_{{2}}^{ - } + {\text{e,}}$$

8 $${\text{2CuCl}}_{{2}}^{ - } + {\text{H}}_{{2}} {\text{O}} \to {\text{Cu}}_{{2}} {\text{O}} + {\text{2H}}^{ + } + {\text{4Cl}}^{ - } ,$$

9 $${\text{Cu}}_{{2}} {\text{O + H}}_{{2}} {\text{O + 4Cl}}^{ - } \rightleftarrows {\text{2CuCl}}_{{2}}^{ - } {\text{ + 2OH}}^{ - } ,$$

10 $${\text{Ni + H}}_{{2}} {\text{O}} \to {\text{NiO + 2H}}^{ + } {\text{ + 2e}}{.}$$

At the beginning of the reaction, Cu element in the alloy is first transformed into CuCl^2−^ due to Cl^−^ in seawater, and then CuCl^2−^ hydrolyzes to produce Cu_2_O. Cuprous oxide has good corrosion resistance and can protect the matrix. Due to the action of Cl ions in seawater solution, the Cu_2_O product film formed will dissolve. The different microstructure composition (Cu/Ni ratio and the addition of other alloying elements) in the WM, BM and HAZ, the bonding strength of the product film is different to some extent. Therefore, under the action of high velocity seawater, the product film will be stripped, resulting in the contact between the matrix and seawater, and aggravate the corrosion reaction. At 1.85 m/s and 3 m/s, the reason why the BM exhibits cathode current within 1–5 h is that the Cu_2_O product film gradually forms to protect the matrix, while between 5 and 8 h, due to the action of chloride ions, Cu_2_O will dissolve, thus reducing the corrosion resistance and gradually turning into anode current. Under the action of 6 m/s high shear force, the products formed by the BM are difficult to stay on the matrix, resulting in accelerated anodic corrosion. At 1.85 m/s and 3 m/s, the HAZ has a series of internal changes due to the effect of thermal stress in the welding process, which is not strong enough to bond with the Cu_2_O corrosion products, so the corrosion resistance is different from that of the BM to some extent. The phenomenon of "large cathode and small anode" will be formed by galvanic corrosion, and the HAZ will be used as the anode to accelerate the corrosion. It should be noted that in the HAZ at 6 m/s, due to high flow rate and high shear force, Ni element and product film fall off very seriously, which will improve the corrosion resistance. Due to the addition of other elements, the weld has higher Ni element. Therefore, the weld film is formed more quickly, more dense and not easy to peel off, so the corrosion resistance is particularly excellent, always as a cathode to slow down corrosion.

## Conclusions

The effect of different flow rates on the electrochemical corrosion of B10 copper-nickel alloy welded joint under flowing seawater has been studied in detail. Different flow rates rendered a significant influence on overall corrosion performance of welded joints, and three zones of welded joints also exhibited obvious corrosion differences under different flow rates. The following conclusions can be drawn from the current results:

1. Under the condition of flow corrosion, the corrosion resistance of BM, HAZ and WM generally increased with the extension of time, and the corrosion rate decreased. The corrosion resistance followed the given trend: WM > BM > HAZ.
2. The corrosion resistance of the BM decreased with the increase of flow rate after 12 h, and excessive Ni element was separated and the product film was spalled under high flow rates, improving the corrosion resistance of HAZ after 12 h. The microstructure of the WM was dense and the components were evenly shed. Hence, the corrosion resistance of the WM was high at low flow rates.
3. In the array electrode test, the WM always acted as a cathode to slow down the corrosion, and the HAZ was transformed into a cathode by polarity deflection at high flow rates. In other states, the HAZ is always the weak link of welding joint corrosion as the anode to accelerate corrosion.
